# Improved single-swab sample preparation for recovering bacterial and phage DNA from human skin and wound microbiomes

**DOI:** 10.1186/s12866-019-1586-4

**Published:** 2019-09-05

**Authors:** Samuel Verbanic, Colin Y. Kim, John M. Deacon, Irene A. Chen

**Affiliations:** 1 Department of Chemistry and Biochemistry, University of California, Santa Barbara, CA USA; 2 Program in Biomolecular Sciences and Engineering, University of California, Santa Barbara, CA USA; 3 Goleta Valley Cottage Hospital, Ridley-Tree Center for Wound Management, Goleta, CA USA; 4 Department of Chemical and Biomolecular Engineering, University of California, Los Angeles, CA USA

**Keywords:** DNA purification, Bacteriophage, Skin microbiome

## Abstract

**Background:**

Characterization of the skin and wound microbiome is of high biomedical interest, but is hampered by the low biomass of typical samples. While sample preparation from other microbiomes (e.g., gut) has been the subject of extensive optimization, procedures for skin and wound microbiomes have received relatively little attention. Here we describe an improved method for obtaining both phage and microbial DNA from a single skin or wound swab, characterize the yield of DNA in model samples, and demonstrate the utility of this approach with samples collected from a wound clinic.

**Results:**

We find a substantial improvement when processing wound samples in particular; while only one-quarter of wound samples processed by a traditional method yielded sufficient DNA for downstream analysis, all samples processed using the improved method yielded sufficient DNA. Moreover, for both skin and wound samples, community analysis and viral reads obtained through deep sequencing of clinical swab samples showed significant improvement with the use of the improved method.

**Conclusion:**

Use of this method may increase the efficiency and data quality of microbiome studies from low-biomass samples.

**Electronic supplementary material:**

The online version of this article (10.1186/s12866-019-1586-4) contains supplementary material, which is available to authorized users.

## Background

The role of the microbiome in human health and disease has become increasingly recognized over the past decade [1]. Methodological improvements in sample processing, sequencing, and bioinformatics are crucial to advance studies of the phylogenetic and functional diversity of the microbes colonizing the human body. The bacterial fraction of the microbiome is often the focus of such studies, revealing associations between bacterial community composition or gene expression and disease states [2]. However, in recent years, the viral component of the microbiome has been gaining attention as well. Particularly notable are viruses that infect bacteria (phages), which may modulate microbial community composition and physiology within the microbiome [3, 4]. While most viral studies have focused on the gut microbiome due to its high microbial load, the skin microbiome is also of high interest for its contribution to dermatological disease states [5, 6]. In addition, the skin is a major site of infection (e.g., in diabetic patients) and thus the microbiome of skin and wounds is relevant to highly morbid diseases. However, analysis of the skin virome is methodologically challenging, due at least in part to the low biomass obtained from typical noninvasive sampling procedures, such as swabbing. Indeed, [7] recently highlighted the need for attention to methodological issues in skin microbiome studies.

Several studies have made progress in optimizing the methodology of skin microbiome studies with a focus on the bacterial composition. Sampling methods of varying invasiveness have been compared (e.g., swab vs. biopsy) [8, 9] and DNA extraction methods and kits have been studied, in particular to improve the representation of Gram-positive organisms through increased physical and chemical lysis [10–12]. Bioinformatic analysis has also been a target for optimization, such as in comparing sequencing methods [10] and variable regions of the 16S rRNA gene to best analyze community composition [13, 14].

Despite this progress, relatively little work has been done to optimize DNA recovery from the phage fraction of the skin microbiome, which presents unique challenges. Sequencing the virome requires shotgun sequencing of genomic DNA preparations due to the lack of conserved genes. Although phages outnumber bacteria in terms of particle number, the small size of their genomes means that typically only a small proportion of the DNA in a sample represents phage genomes [15]. Thus, viral sequencing depth is often limited in low biomass samples, inhibiting downstream bioinformatic processing for contig assembly, community recapitulation, and functional annotation [16]. In addition, use of whole metagenome sampling cannot discern between reads associated with virus-like particles (VLPs) and those associated with lysogenic phages that are integrated into their hosts’ genomes as prophages [17]. A common solution to this problem is to purify the VLPs, i.e., separate VLPs from other microbiota, ultimately enabling greater viral sequencing depth and discernment of genomes associated with VLPs from prophages. Methods have been developed and characterized for VLP purification from high-biomass samples like feces [17] or collected from large volumes of dilute sample, such as seawater [18]. Also, most VLP purification methods require a separate sample to be used for characterizing the bacterial fraction, thus introducing a source of variation into the analysis if one wishes to characterize the virome simultaneously with the bacterial fraction. Clinical scenarios may also hamper collection of multiple replicate samples. Therefore, while these methods can be applied to skin microbiome samples [6], there is nevertheless a need to develop and characterize streamlined protocols maximizing the yield of DNA from VLPs obtained from typical clinical skin and wound samples.

Here we detail a methodology, modified from previous work [6, 18], for swabbing and sample processing that utilizes fractionation and extraction to simultaneously produce both viral-enriched (VLP) and whole-metagenomic samples from a single, low-biomass human skin or wound swab. Modifications include reserving the swab tip and pellet after centrifugation, utilizing more lysis methods, and adjusting reagent concentrations and volumes. We quantify viral DNA enrichment and total viral and bacterial DNA yields in model samples using this method. We also use this procedure to process clinical samples, demonstrating improved recovery of phage DNA for downstream sequencing and characterization.

## Results

### Recovery of phage and bacterial DNA released from swabs

In this procedure, material from a single swab is separated into a VLP fraction and ‘remainder’ fraction by centrifugation (Additional file 1: Figure S1). In brief, the VLP fraction is treated with DNase I to digest free DNA, VLPs are precipitated, and capsids disrupted by sodium dodecyl sulfate (SDS) and proteinase K. VLP nucleic acids are purified by exposure to cetyltrimethylammonium bromide (CTAB), phenol-chloroform extraction, and ethanol precipitation. For the remainder fraction and for unfractionated samples, cells are disrupted by chemical and physical lysis and DNA recovered using a commercial kit.

To better understand the recovery of phage and bacterial DNA from a skin or wound swab, we use mock samples of known concentration, composed of M13 phage and an F- strain of *E. coli* (i.e, non-host strain). Phage and cells were mixed together in a 19:1 ratio and diluted to approximate typical phage:cell ratios and concentrations from human and environmental samples [19]. In addition to DNA extraction itself, there are two major possible points of loss of material: (1) incomplete release of material from the swab, and (2) low removal of material from skin by swabbing. To first address point (1), we applied the mock sample directly to the swab, obtained the VLP and remainder fractions, and measured the yield of DNA recovered. Unfractionated samples were also analyzed as a control.

The amount of M13K07 phage and bacterial DNA extracted from each fraction (VLP and remainder) and from the unfractionated sample were determined by quantitative polymerase chain reaction (qPCR). Recoveries (*r*) are expressed as a fraction of the known total quantity of phage (*r*_p_) and bacteria (*r*_b_) in the entire mock sample (e.g., for an unfractionated sample, complete recovery of bacterial or phage DNA corresponds to *r*_b_ = 1 and *r*_p_ = 1, respectively). To a first approximation, we expect that, upon fractionation by non-equilibrium centrifugation, all bacteria are pelleted in the remainder fraction (expected *r*_b_ = 1 in the remainder and *r*_b_ = 0 in the VLP fraction), while phages remain evenly dispersed in solution (i.e., since the VLP and remainder fractions are equal volume, expected *r*_p_ = 0.5 in both fractions). We also define yield of bacteria (*y*_b_) and phage (*y*_p_) as the ratio of the observed recovery to the expected recovery, expressed as a percentage.

**Fig. 1:**
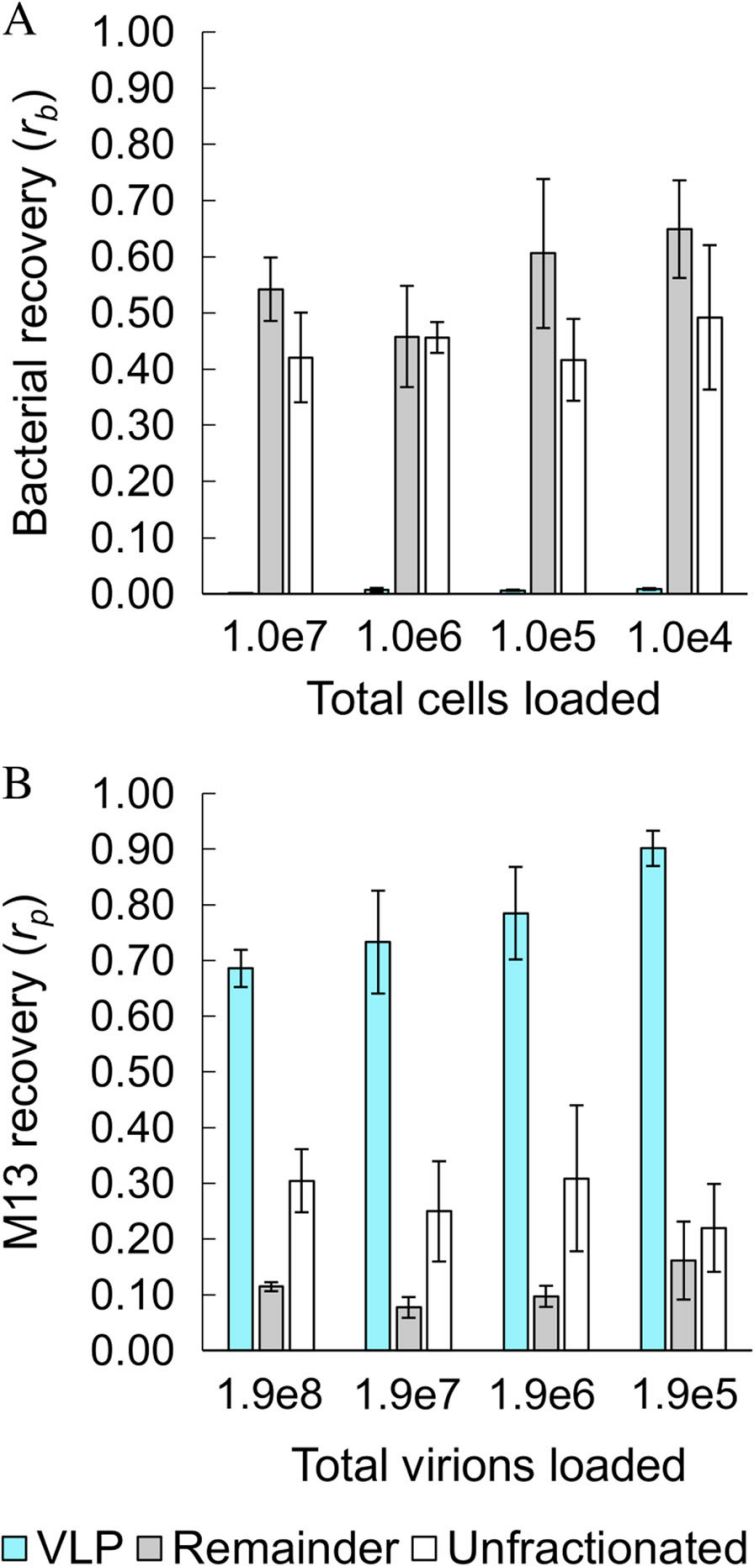
Recovery of bacterial (**a**) and phage (**b**) DNA from mock samples loaded onto swabs in varying amounts. Shown are recoveries for the VLP fraction (cyan), remainder fraction (gray), and unfractionated (white) samples, as determined by qPCR. Negative controls for qPCR containing no template were not quantifiable after 40 cycles of PCR. Error bars indicate standard deviation among biological triplicates

Without fractionation, the recovery of bacterial DNA (*r*_b_ = 0.45 ± 0.04 (*y*_b_ = 45%)) and phage recovery (*r*_p_ = 0.27 ± 0.04 (*y*_p_ = 27%)), across sample loads, indicated that recovery for phage was somewhat less efficient than for bacterial DNA (Fig. 1). For fractionated samples, in the remainder fraction, *r*_b_ = 0.56 ± 0.08 (*y*_b_ = 56%) and *r*_p_ = 0.11 ± 0.04 (*y*_p_ = 22%) across sample loads, similar to yields from the unfractionated samples. In the VLP fraction, *r*_b_ = 0.006 ± 0.003 (*y*_b_ is undefined given an expectation of 0% recovery) while *r*_p_ = 0.78 ± 0.09 (*y*_p_ = 160%) across sample loads (Fig. 1). The apparent phage yield over 100% in the VLP fraction, corresponding to unexpected enrichment in the supernatant, may be due to inaccuracies in quantitation of the stock phage concentration (e.g., conversion factors do not account for compositional or structural irregularities of the phage). Such biases do not affect comparisons of yields between unfractionated samples and remainder and VLP fractions. In the VLP fraction, the ~ 85-fold decrease in cell DNA recovery and ~ 5-fold increase in phage DNA recovery, compared to unfractionated or the remainder fraction, indicates a substantial ~ 400-fold enrichment of DNA recovery (in terms of genome copies) from phages compared to cells. If sequenced, this enrichment would translate into a similar enrichment of phage DNA reads. The overall yields also indicate that DNA from roughly half or more of the phages and cells loaded onto a swab can be recovered in this protocol.

### Amount of DNA released from swabs

**Fig. 2:**
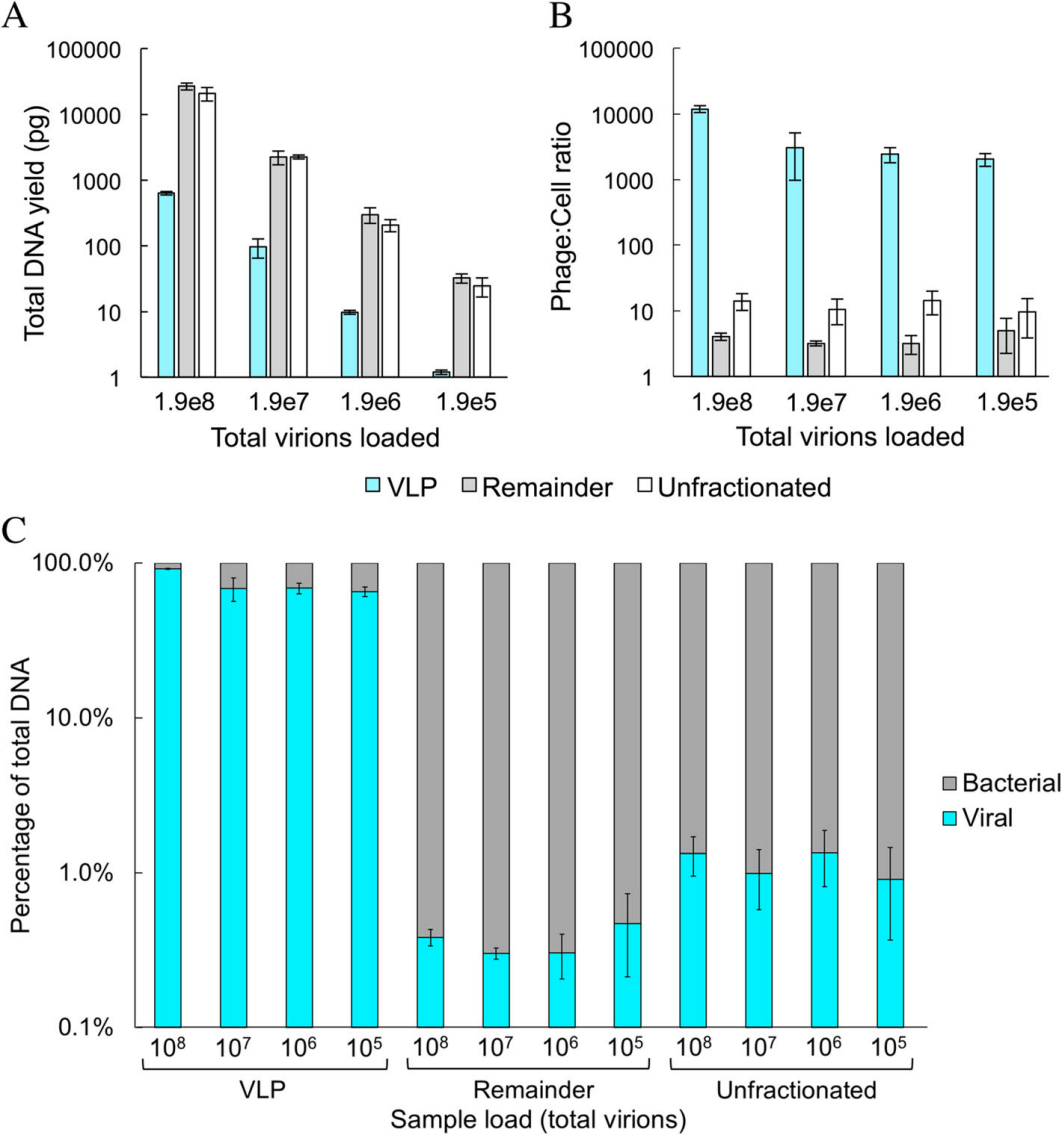
Mass of DNA recovered from swabs loaded with different amounts of M13 and cells. Total (phage + bacterial) DNA mass recovered as determined by Qubit (**a**), recovered phage:cell ratio (**b**) and DNA composition by mass (**c**) in different fractions, as determined by qPCR. Negative controls for qPCR containing no template were not quantifiable after 40 cycles of PCR. Error bars indicate standard deviation among biological triplicates

Since this protocol is intended to produce samples for high-throughput sequencing, total recovered mass is an important metric. Depending on the manufacturer’s instructions, shotgun sequencing library preparation begins with 0.01–10 ng per sample. Total recovered DNA mass in the VLP fractions (phage + bacterial, as determined by qPCR) ranged from 0.63 ± 0.04 ng (from sample originally containing 1.9 × 10^8^ virions) down to 1.2 ± 0.09 pg (from sample originally containing 1.9 × 10^5^ virions) (Fig. 2a). The concentration of VLP fractions is sufficient for low-input metagenomic library preparation without amplification, with the exception of the 1.9 x 10^5^ virion sample, which was expected to yield insufficient DNA. From the remainder fractions, the total genomic DNA (gDNA) mass ranged from 27 ± 3 ng (from sample originally containing 10^7^ cells and 1.9 × 10^8^ virions) down to 32 ± 5 pg (from sample originally containing 10^4^ cells and 1.9 × 10^5^ virions), which is adequate for low-input metagenomic library preparation and 16S rRNA sequencing (Fig. 2a). Thus, swabs containing samples in this concentration range yield sufficient DNA for bacterial analysis, and may yield sufficient DNA for phage analysis if the swab contains at least ~ 10^6^ virions (depending on the phage).

The number of phage genome reads obtained from sequencing a VLP sample depends not only on the relative enrichment of VLP DNA compared to cell DNA, but also on the relative genome sizes. Since bacterial genomes are 10–1000 times larger than phage genomes, if no enrichment is performed, reads from bacterial genomes typically vastly outnumber reads from phage genomes. Indeed, without fractionation, M13K07 DNA represented 1.1 ± 0.5% of the total DNA by mass across sample loads (Fig. 2c), consistent with expectation for the initial sample (1.8%, based on *E. coli* genome size: 4.56 MB [20], M13 ssDNA genome size: 8669 nt, and 19:1 phage:bacteria ratio) and the somewhat lower recovery of phage compared to bacterial DNA.

In contrast, in VLP fractions, M13K07 DNA represented 73 ± 13% of the mass of recovered DNA across sample loads (Fig. 2c), corresponding to a 67-fold increase, on average, in the proportion of phage DNA out of total DNA, compared to the unfractionated samples. In a metagenomic sequencing sample, this would correspond to a similar increase in the fraction of reads from phage DNA. In terms of the apparent phage:cell ratio based on recovered DNA, which was approximately 12:1 in the unfractionated samples, fractionation enriched the VLP fraction to an apparent phage:cell ratio of ~ 2000:1 to ~ 12,000:1 (Fig. 2b).

### Limit of detection of phage swabbed from human skin

**Fig. 3:**
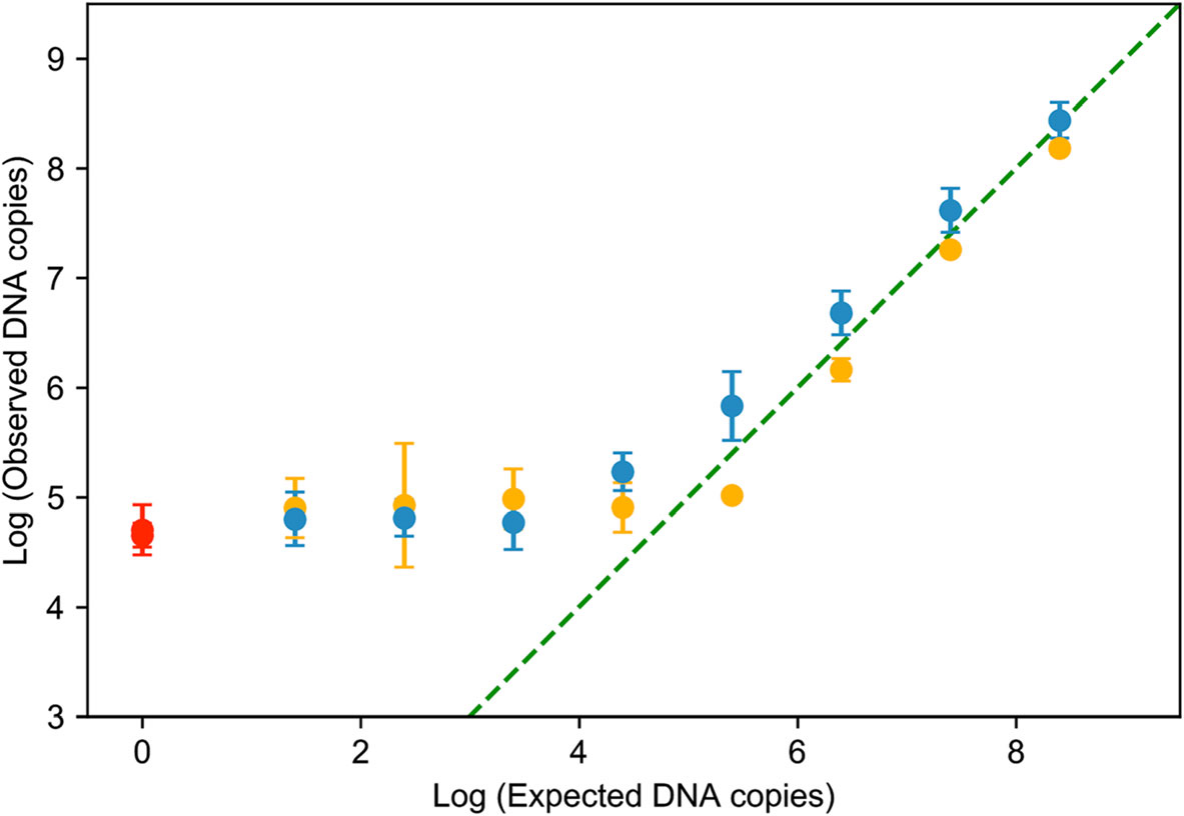
Quantitative recovery of M13 loaded onto skin and then swabbed immediately (blue) or after drying (orange), as determined by qPCR. Expected or observed DNA copies per swab is indicated. Complete recovery is indicated by the dotted green line. A negative control (red) indicates experimental background determined by loading of 1X TE onto skin followed by swabbing and qPCR, and therefore reflects the combined background from skin, swabbing and extraction, and qPCR reagents. Error bars indicate standard deviation among biological triplicates

Having validated the method using phage:cell mixtures placed directly onto swabs, we moved to determine recovery of DNA when including the second potential source of loss, swabbing from human skin. An M13KO7 phage stock was serially diluted ten-fold and samples were loaded onto human skin, then swabbed immediately or allowed to dry prior to swabbing. Swabs were processed analogously to the above experiments. Near quantitative yield was obtained, for samples in which ~ 10^5^ or more virions were loaded onto the skin (Fig. 3). Lower sample loads than this could not be distinguished from qPCR background. Wet samples were observed to have consistently higher yields than dry samples; this phenomenon may be due to denaturation of phage upon drying and was also observed for T4, which showed a pronounced decrease in recovery for dried samples (see next section). The limit of detection of ~ 10^5^ virions corresponds to ~ 400 fg of ssDNA.

### Recovery of T4 and bacterial DNA from skin swabs

**Fig. 4:**
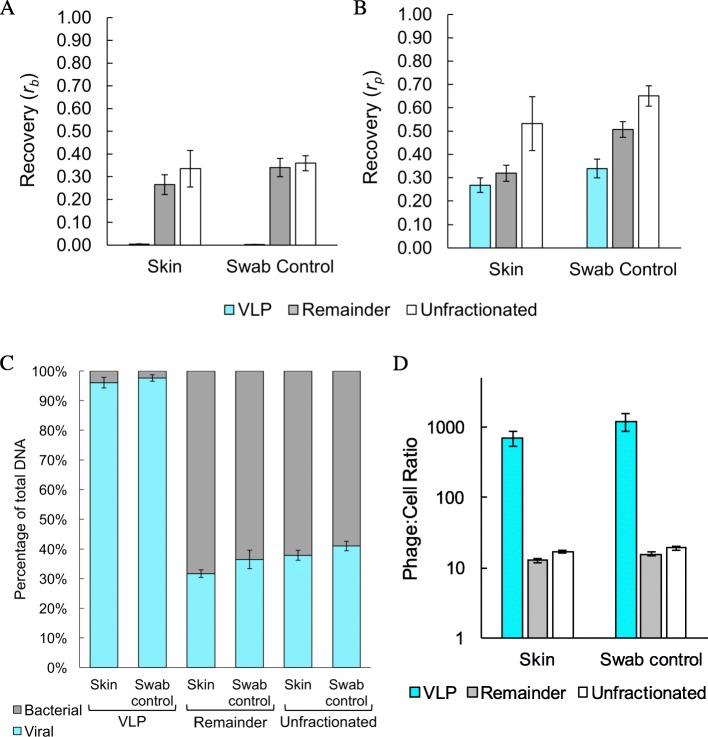
Recovery and mass yield from mock skin and swab samples with phage T4. Bacterial (**a**) and phage T4 (**b**) DNA recoveries were determined by qPCR for a single sample load (1.0 × 10^8^ virions and 1.0 × 10^7^ cells). Negative controls for qPCR containing no template were not quantifiable after 40 cycles of PCR. DNA composition (**c**) and phage:cell ratios (**d**) as determined by qPCR characterize T4 enrichment in the VLP fraction. Error bars indicate standard deviation among biological triplicates

To test the compatibility of other phage morphologies with this method, analogous experiments were performed using the canonical *Caudovirales* phage T4, in place of M13K07, for a skin swabbing experiment. A Δ*ompC* Δ*ompF* strain of *E. coli* was selected for this experiment to avoid the confounding effect of phage adsorption and infection. T4 and *E. coli* were titered spectrophotometrically and mixed in a 10:1 ratio (10^8^ virions: 10^7^ cells), loaded onto skin, then swabbed immediately while wet. DNA recovery values were comparable to the M13 experiment. In the remainder fraction, phage recovery *r*_p_ = 0.32 ± 0.03 (*y*_p_ = 64%) and bacterial recovery *r*_b_ = 0.26 ± 0.04 (*y*_b_ = 26%) were similar to unfractionated samples (*r*_p_ = 0.53 ± 0.12 and *r*_b_ = 0.34 ± 0.08) (Fig. 4a,b). In the VLP fraction, phage recovery *r*_p_ = 0.27 ± 0.03 (*y*_p_ = 54%) and bacterial recovery *r*_b_ = 0.004 ± 0.001 (*y*_b_ is undefined) indicated enrichment of phage, as expected. Controls in which phage and cells were applied directly to the swab showed similar recoveries, consistent with expectation given near quantitative yield from swabs.

Total phage DNA mass recovered is substantially higher than for M13KO7, consistent with the larger genome of T4, with the amount of DNA recovered from the VLP fraction being 5.1 ± 0.6 ng, and DNA recovery from the remainder fraction being 18 ± 3 ng on average. These amounts are more than sufficient for typical next-generation sequencing (NGS) preparation protocols.

The recovered DNA from the VLP fraction was composed of 96 ± 1% T4 DNA by mass, a substantial increase compared to the unfractionated control (32 ± 2%) (Fig. 4c). This increase is less dramatic than for M13K07, due to the larger genome size of T4. Apparent phage:cell ratios after recovery also indicate significant viral enrichment, as fractionation resulted in a phage:cell ratio of ~ 700:1 in the VLP fraction, compared to that of unfractionated controls (~ 17:1) (Fig. 4d).

Swabbing was also performed from dried T4 samples, but these were found to produce very low yields in the VLP fraction compared to the analogous M13K07 experiment. However, the remainder fraction of the dried samples gave T4 DNA amounts comparable to swab controls, indicating that dried T4 could be recovered from the skin but was lost in the VLP purification process. We hypothesized that this was due to capsid damage that occurred during desiccation on the skin, which then exposed phage DNA to DNase digestion and thus reduced DNA purified in the VLP fraction. A plaque-forming assay was performed to determine the concentration of viable phage particles after desiccation; indeed, the VLP fraction from dried T4 produced ~ 100-fold fewer plaques than the VLP fraction of a wet T4 sample.

### Recovery of bacterial and phage DNA from clinical wound and skin swabs

**Fig. 5:**
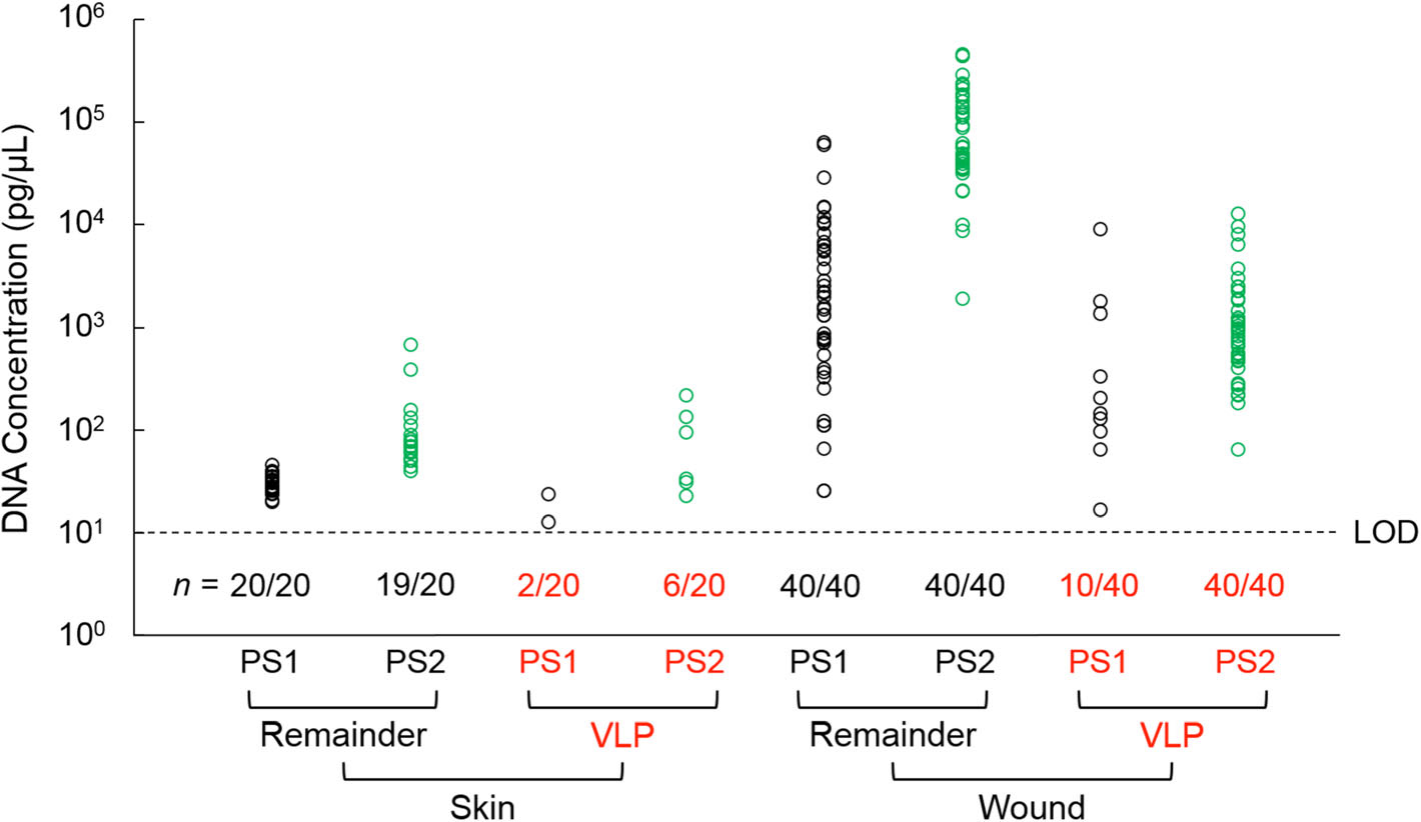
Comparison of DNA yields from clinical skin and wound swabs using kit-based extraction (PS1) and the method described here (PS2). DNA was quantified fluorometrically by Qubit assay. Limit of detection is indicated by the dashed line (LOD for the Qubit dsDNA HS assay = 10 pg/μL according to supplier documentation [21]). The fraction of samples above the LOD (*n*) is listed. All negative controls, which were exposed to air in the collection room or blank extractions, were below the LOD

To test whether this processing method improved recovery of phage DNA from clinical swab samples, swabs were obtained from normal skin and wounds collected from patients at a wound clinic and processed in preparation for high throughput sequencing. We compared the novel sample preparation method (pilot study 2, or PS2) to a standard kit-based extraction method (pilot study 1, or PS1) by measuring dsDNA yields fluorometrically. Using PS1, only 10% of skin VLP fractions and 25% of wound VLP fractions yielded detectable DNA. However, PS2 gave significant improvement, producing detectable DNA in 30% of skin VLP samples and 100% of wound VLP samples (Fig. 5). Of VLP samples containing a detectable amount of DNA, PS2 yielded 6.7- and 4.4-fold greater average DNA concentration for skin and wound swabs, respectively, compared to PS1. Remainder fractions, which include substantial bacteria, are expected to contain more DNA, and as expected, nearly all skin and wound samples produced a detectable amount of DNA in the remainder fraction. In addition, PS2 gave 3.9- and 16.6-fold higher average DNA concentration compared to PS1, indicating that PS2 would also improve DNA yield for whole metagenome studies.

**Fig. 6:**
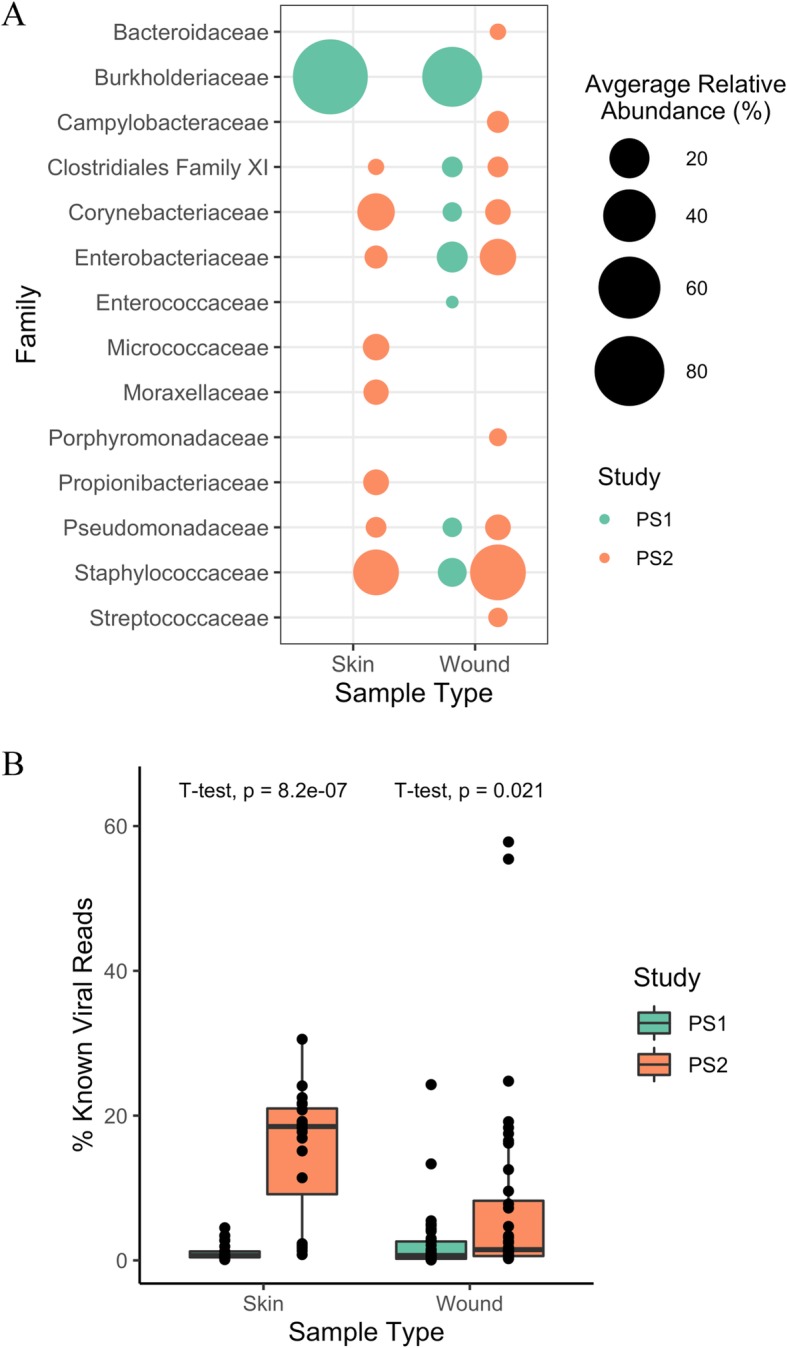
Comparisons of DNA composition from clinical skin and wound swabs using kit-based extractions (PS1) and the method described here (PS2). For the remainder fraction, bacterial community composition was deteremined by 16S rRNA sequencing (**a**). Composition was summarized by averaging the relative abundance of taxa at the Family level across sample type (skin or wound). Families with > 2% average relative abundance are shown. Recovery of viral DNA in the VLP-enriched fraction was estimated by shotgun sequencing and read mapping to the IMG/VR viral metagenome database (**b**). Percentage of reads mapped per sample are plotted here, and mean percentages are compared by *t*-tests

To assess the quality of the extracted DNA, samples from both studies were sequenced by paired-end Illumina MiSeq. Bacterial composition of the remainder fractions was determined by 16S rRNA sequencing using the V1-V3 loops (Fig. 6a). Both skin and wound samples from PS1 were largely dominated by *Burkholderiaceae*, a well known kit contaminant [22, 23]. However, PS1 wound samples also contained low levels of previously reported skin colonizers such as *Corynebacteriaceae*, *Staphylococcaceae*, and *Pseudomonadaceae* [24]. In contrast, PS2 skin and wound samples did not suffer from the same apparent kit contamination as PS1, and PS2 samples appear to contain archetypal skin and wound microbiomes. On average, the most abundant PS2 skin community members were commensals and opportunists, including *Corynebacteriaceae, Staphylococcaceae, Proprionibacteriaceae,* and *Micrococcaceae* [24, 25]*.* PS2 wound samples had high levels of *Staphylococcaceae* and *Enterobacteriaceae,* as well as lower levels of other previously reported wound colonizers like *Bacteroidaceae, Campylobacteriaceae, Clostridiales, Porphyrmonadaceae, Pseudomonadaceae,* and *Streptococcaceae* [26–28]*.* These findings confirm that the novel fractionation and extraction protocol produces high quality DNA sufficient for sequencing, resulting in improved community recapitulation compared to the kit-based extraction used here.

VLP-enriched samples were shotgun sequenced, and the quantity of recovered viral DNA was estimated by mapping quality-controlled reads to the Joint Genome Institute’s integrated microbial genomes viral analysis (IMG/VR) metagenomic database (Fig. 6b). On average, only 1.1 ± 1.2% of PS1 skin reads and 2.2 ± 4.3% of PS1 wound reads mapped to the database. PS2 samples had significantly higher viral mapping rates, with averages of 15.2 ± 8.9% for skin samples and 7.5 ± 13.2% for wound samples, which corresponded to higher absolute number of known viral reads (Additional file 1: Figure S2A). However, PS2 samples also had higher levels of human DNA contamination (Additional file 1: Figure S2B). Although the IMG/VR database is likely largely incomplete, these results show that the novel method produces more known viral reads on average.

## Discussion

As microbiome studies advance, there is increasing interest in the relatively understudied virome. However, experimental methodology for virome sampling has not been characterized and optimized as extensively as methods for bacterial sample processing. A central issue is the low biomass of phages in samples obtained from skin and wounds, which leads to insufficient material for sequencing or inadequate sequencing depth. Previously reported methods tend to be lengthy, not optimized for small volume, low-biomass samples, and/or do not retain the non-VLP fraction of the microbiome, requiring acquisition of a second sample for virome and whole microbiome analysis. These factors limit practical usage in a clinical setting.

Here, we describe a streamlined method for preparing both viral-enriched and whole microbiome samples from a single, low-biomass skin or wound swab. Using this method, we produce VLP-enriched samples with several hundred-fold enrichment of viral DNA and also retain a whole microbiome fraction. Both fractions typically yield sufficient quantity of DNA for sequencing library preparation. This method may provide the following practical benefit in a clinical setting. In addition to only requiring one swab, in comparison to traditional methods (PS1), in which the majority of skin and wound VLP samples did not yield sufficient DNA for further analysis, the technical improvements presented here (PS2) produce sufficient VLP DNA from 100% of wound swabs and 30% of skin swabs. The increased yield from this protocol is likely due to the accumulation of small improvements. For example, buffer concentrations were chosen to facilitate replicable pipetting and reduce total sample volume, allowing precipitation and extraction steps to be performed in microcentrifuge tubes and a single phase-lock gel tube, thus minimizing loss from transfers. Additionally, we find that the use of ethanol precipitation for VLP DNA purifiaction is more efficient than column-based purification. Most of the buffers are not available off-the-shelf in the concentrations used here, but are easily prepared and stored as such. The PS2 workup requires more time at the bench, but is less costly in terms of reagents in addition to consistently producing higher DNA yields than PS1, in turn producing higher quality 16S rRNA and metagenomic sequencing data.

This protocol was developed with the goal of capturing free ssDNA and dsDNA phages, so mock specimens were chosen accordingly to characterize the protocol (M13 and T4, respectively). These phages also represent a number of clinically important phages. As a member of *Inoviridae*, M13 is a relative of the clinically implicated *Pseudomonas aeruginosa* phage Pf [29]. T4 is a member of the canonical dsDNA *Caudovirales* family, which have previously been shown to be prominent members of the healthy skin virome [6]. We noted that T4 survived poorly on the dessicated environment of the skin compared to M13; this may or may not be relevant depending on whether a study is intended to survey the viable phages. However, an important caveat of the present work is that the procedure is not optimized for lipid-encapsulated or RNA viruses. Additionally, in mock samples, phage:cell stock solutions were only prepared in an approximately 10:1 ratio, and variations of this ratio were not tested. Nevertheless, the clinical samples presumably varied in phage:cell ratio and other factors.

Although only a small proportion (typically < 1%) of the total bacterial DNA was found in the VLP fraction, the large molecular weight of bacterial DNA translates into a large mass fraction relative to phage DNA. This effect is pronounced in the case of M13, a very small phage (~ 8 kb ssDNA), for which ~ 70% of the VLP fraction consisted of phage DNA. We anticipate that ~ 70% thus represents a lower bound on the mass fraction of phage DNA in realistic settings where multiple viral species are recovered. As expected, the effect is less noticeable in the case of T4 (~ 123 kb dsDNA). Thus, despite the lack of lengthy purification steps (e.g., CsCl gradient centrifugation), most reads from a sequencing run using the VLP DNA would be expected to derive from phages.

We applied this method in the clinic and found that it dramatically reduced the amount of reagent and consumable contaminants detected in 16S rRNA sequencing data. Additionally, the method produced VLP-enriched samples with higher viral read mapping rates than the kit-based extractions. However, the viral read mapping rates were still relatively low, which is likely due to high human DNA contamination and an incomplete viral reference database. We hypothesize that the high level of human DNA contamination was due to increased DNA yields and insufficient DNase I digestion, which can be remedied with higher nuclease concentrations and increased incubation times. While IMG/VR is the most comprehensive viral metagenome database available [30], database matching is insensitive to novel viruses, which could account for a large proportion of the DNA; it is estimated that only 10–60% of viral metagenomes align to reference databases [31, 32]. Use of this protocol could have several benefits for virome and microbiome studies, including increased patient recruitment due to minimal swabbing, improved experimental design using paired VLP and remainder fractions, reduced failure rate of DNA extraction from individual swabs, and improved detection of low abundance phages.

## Conclusions

With rapid advancements in microbiome studies, the frequently overlooked virome is gaining interest. However, investigation of healthy and diseased human viromes from whole metagenome samples can be hindered by low-biomass samples. To overcome these challenges, VLP purification methods have been developed. However, previously reported methods are not compatible with small volume, low-biomass samples and do not retain the non-VLP fraction of the microbiome, requiring separate samples for virome and whole microbiome analysis. Here, we describe a method for preparing both viral-enriched and whole microbiome samples from a single, low-biomass skin swab to facilitate the study of viromes and microbiomes in dermatological diseases. Using this method, we produce VLP-enriched samples composed of > 70% viral gDNA with up to 80% yield of viral gDNA and less than 1% yield of bacterial gDNA. The remaining whole microbiome fraction is retained in this process, allowing paired VLP and whole metagenome sequencing. In a clinical setting, this method improves both microbial community analysis as well as the number of viral reads, demonstrating that technical improvements can significantly impact the quality of sequencing data from low-biomass samples.

## Methods

### Phage and bacterial sample preparation

Mock samples were composed of M13KO7 phage and BL21(DE3) *E. coli* (NEB C2527), or T4 phage and KJ740 *E. coli* (CGSC 12151). The virion density of phage stocks was determined spectrophotometrically as follows. Absorbance was measured at 269 nm and 320 nm and converted to virions/mL by the following equation: virions/mL = [(A_269 nm_ - A_320 nm_) x (6 × 10^16^)]/genome length (8669 bp for M13KO7, 168.903 kb for T4) [33]. 5 mL cultures of *E. coli* were inoculated by a single colony and grown in Luria broth (LB) overnight in the presence of 100 μg/mL ampicillin for strain BL21(DE3) or 10 μg/mL tetracycline for strain KJ740. Cell density of *E. coli* overnight cultures was measured by optical density at 600 nm (OD_600_) with 4- and 10-fold dilutions, and converted to cells/mL using a conversion factor of 3.2 × 10^8^ cells/mL per OD_600_ unit. This conversion factor was experimentally determined by comparing OD_600_ measurements and colony-forming unit (CFU) counts of overnight cultures. Known concentrations were used to create a mixed phage and cell stock solution in a 19:1 ratio, for final concentrations of 9.5 × 10^6^ virions/μL and 5.0 × 10^5^ cells/μL, in 0.5X TE, 0.5X LB (phage stock was diluted in 1x TE and cells were diluted in LB). For M13KO7 viral enrichment experiments, the mixed stock was 10-fold serially diluted 3 times in LB to create a sample range of 9.5 × 10^5^–9.5 × 10^3^ virions/μL (5.0 × 10^4^–1.0 × 10^2^ CFU/μL), and 20 μL of each dilution was loaded directly onto a sterile swab. In the T4 viral enrichment experiment, one mixed stock was created at 5.0 × 10^6^ virions/μL (5.0 × 10^5^ CFU/μL).

### Swabbing from human skin

All swab experiments were performed with sterile Copan FLOQSwabs 520C. Three healthy volunteers were enrolled after obtaining informed consent according to procedures approved by the UCSB Human Subjects Committee and Institutional Review Board (Protocol 4-18-0190 and 2-18-0059). M13KO7 phage alone was diluted serially in 1X sterile TE*,* for a concentration range from 1.9 × 10^8^ to 1.9 × 10^2^ virions/μL. For skin sampling experiments, skin on the left forearm of the volunteer was wiped with 70% ethanol. Then, sample was applied to the skin (20 μL for T4 experiments or 10 μL for M13K07 experiments), covering an area of approximately 0.25 cm^2^, and either swabbed immediately or allowed to dry for 30 min. The swab was pre-wetted with sterile 1X TE and then rotated 10 times over a ~ 1 cm^2^ area (length of the swab tip = 1 cm) with gentle pressure (Levine’s technique). The yield of phages and bacteria recovered from such a swab reflects two steps: gathering of material from the skin, and release of material from the swab. To separately quantify the yield of phage and cells released from the swab, the same volume of the stock dilution series was applied directly to a different swab. Swabs containing sample were processed further within 30 min. Negative controls were performed by pipetting an equal volume of 1X TE onto the skin and following the procedure outlined above.

### Fractionation of virus-like particles (VLPs)

After swabbing, the swab tip was inserted into a 1.5 mL microcentrifuge tube and snapped at the 30 mm breakpoint. 500 μL of sterile 1X TE was added to the tube, and the tube was vortexed for 2 min at maximum speed on a multitube vortex adapter to resuspend the sample. Samples were then centrifuged at 16,000 x g for 2 min to pellet cells. 250 μL of supernatant was transferred to a 2 mL microcentrifuge tube for immediate VLP precipitation (the VLP fraction). The remaining 250 μL of supernatant, pelleted cells, and swab tip (the ‘remainder’ fraction) were kept in the original tube and stored at − 20 °C before proceeding to DNA extraction.

### Isolation of DNA from virus-like particles

To digest free DNA in the VLP fraction, 2 μL of 126x DNase I reaction buffer (441 mM MgCl_2_, 63 mM CaCl_2_) and 2.5 μl DNase I (5 units, NEB) were added to the VLP fraction, mixed by inversion, and incubated at 37 °C for 30 min. DNase I was inactivated by incubation at 75 °C for 10 min. VLPs were precipitated by adding 25 μL sterile 1X TE (pH 8.0), 2.5 μL 0.5 M EDTA (pH 8.0), 250 μL formamide, 7 μL glycoblue (15 mg/mL), and 1.1 mL 100% ethanol, followed by incubation at − 20 °C for 1 h and centrifugation for 1 h at > 10,000 x *g* at 4 °C. Pellets were washed with 500 μL of ice cold 70% ethanol and re-pelleted by centrifugation for 30 min at > 10,000 x *g* at 4 °C. Pellets were dried for 1 h at room temperature in a vacufuge before being resuspended in 152 μL sterile 1X TE (pH 8.0).

Viral capsids were disrupted and digested by adding 19.6 μL of 10% SDS and 21.4 μL proteinase K (20 mg/mL) to the resuspended VLPs, followed by incubation at 55 °C for 1 h. Then, 32 μL of 5 M NaCl and 25 μL CTAB-NaCl were added followed by incubation at 65 °C for 10 min. The 250 μL sample was then transferred to a phase lock gel tube (5PRIME PLG Light) and mixed with 250 μL of 25:24:1 phenol:chloroform:isoamyl alcohol by inversion. Phases were separated by centrifugation at 1500 x *g* for 5 min. In the same tube, 24:1 chloroform:isoamyl alcohol extraction was performed twice and centrifuged as described above, and the 250 μL aqueous phase was transferred to a 2 mL microfuge tube. DNA was precipitated by adding 27.5 μL of 3 M sodium acetate (pH 5.2), 1 μL Glycoblue (15 mg/mL), and 1.5 mL 100% ethanol followed by incubation at − 80 °C for 1 h and centrifugation at > 13,000 x *g* at 4 °C. Pellets containing DNA were washed with 500 μL ice cold 70% ethanol, centrifuged at > 13,000 x *g* at 4 °C for 30 min, dried for 1 h at room temperature in a vacufuge, and resuspended in 20 μL 1X TE (pH 8.0).

### Extraction of genomic DNA from the remainder fraction and unfractionated samples

Samples were thawed on ice, then vortexed for 2 min at maximum speed using a multitube vortex adapter to resuspend the cells. Keeping the swab in the tube, lysis was performed by adding 5 μL of 5 M NaCl and 45 μL of Ready-Lyse lysozyme (250 U/μL) (Epicentre), followed by incubation at room temperature for 30 min. Lysis continued with addition of 33 μL of proteinase K (20 mg/mL) and 333 μL of PureLink Genomic Lysis/Binding buffer (Thermo), followed by incubation at 55 °C for 1 h. Physical lysis was conducted with bead beating by adding 2 g of 0.5 mm glass beads to the sample and vortexing at maximum speed for 10 min using a multitube vortex adapter. 333 μL of 100% ethanol was added to the sample and mixed by inversion, and the entire sample was loaded onto a PureLink Genomic DNA Mini Kit column (Thermo). DNA cleanup and elution was performed using the kit’s guidelines, with an elution volume of 25 μL EB.

### Collection, processing, and quantification of clinical samples

Clinical sample collection was performed at Ridley-Tree Center for Wound Management at Goleta Valley Cottage Hospital in accordance with protocols approved by the Cottage Health Institutional Review Board (Study Protocol 16-52u and 17-48u). We recruited a cohort of 40 wound care patients and collected samples after obtaining informed consent from the patient. Exclusion criteria were: patients under the age of 18, in the intensive care unit, or presenting with an unrelated non-wound infection. Four clinically classified chronic wound types were sampled (diabetic, venous, arterial, and pressure ulcers), with ten patients per wound type. Wound swabs were collected pre- and post-debridement, and a healthy skin swab was collected from the contralateral limb. Negative control samples were collected by exposing swabs to air in the collection room for the same duration as wound and skin swab collection. All swabs were collected using Levine’s technique as described above for the mock samples. Swabs were placed back into the dry, sterile collection tube and stored at 4 °C for no more than four hours before being processed.

To determine whether the fractionation and purification procedures described above affected DNA yield from swabs obtained in a clinical setting, samples from 20 patients (five patients per wound type; designated as PS1 samples) were processed using a standard processing protocol, while samples from the other 20 patients (five patients per wound type; designated as PS2 samples) were processed using the fractionation and extraction methods described in detail above. The standard processing protocol for PS1 is as follows: 500 μL of sterile 1x TE was added to the collection tube and vortexed for 2 min at maximum speed to resuspend the sample, which was then transferred to a microcentrifuge tube and centrifuged at 16,000 x *g* to pellet cells, then 250 μL of supernatant was filtered using a 13 mm 0.45 μm polyethersulfone syringe filter to produce the VLP fraction while the remaining 250 μL supernatant and pellet constituted the remainder fraction. VLP fractions were subjected to DNase I treatment as described above, then extracted using the PureLink Viral RNA/DNA Mini Kit following the manufacturer’s instructions with an elution volume of 25 μL. The remainder fraction was extracted using the PureLink Genomic DNA Mini Kit following the manufacturers instructions with an elution volume of 25 μL. DNA yields were quantified fluorometrically using the Qubit dsDNA High Sensitivity kit on the Qubit 3 instrument, with 5 μL of sample used per assay.

### Quantitative PCR

To generate stocks for qPCR standard curves, M13KO7 and T4 virion DNA was extracted using the PureLink Viral RNA/DNA Mini Kit and *E. coli* gDNA was purified using the PureLink Genomic Mini Kit. Stock concentrations were determined by Qubit ssDNA and dsDNA High Sensitivity reagents. Stocks were then diluted in sterile 1X TE to create 10-fold dilution series with the following concentration ranges: 1.8 × 10^9^–18 copies/μL for M13K07; 1.7 × 10^7^ to 17 copies/μL for T4; 1.1 × 10^6^ to 11 copies/μL for BL21(DE3) *E. coli*; 1.3 × 10^7^ to 13 copies/μL of KJ740 *E. coli*. qPCR primers were designed on Benchling using Primer3 and were purchased from Integrated DNA Technologies, generating 100 bp amplicons for M13, T4, and *E. coli*. Primer sequences used: M13K07 Forward 5′-TCTGTACACCGTTCATCTGTCC-3′, M13K07 Reverse 5′-ACCTGCTCCATGTTACTTAGCC-3′; T4 Forward 5′-AGCGACCCGGTTTCTCATTT-3′, T4 Reverse 5′-AAATTACGTCCCGCTGGTGT-3′; 16S rDNA V1 Forward 5′-ATTGAACGCTGGCGGCAGG-3′, 16S rDNA V1 Reverse 5′-CCCAGACATTACTCACCCGTCCG-3′. qPCR experiments were performed using Bio-Rad SsoAdvanced Universal SYBR Green Supermix and Bio-Rad CFX96 thermal cycler with CFX96 Real-Time PCR Detection System. Final volume of the reaction was 20 μL, containing 10 μL of SYBR Green Supermix, 1 μL of each primer at 10 μΜ, 1 μL template, and 7 μL of PCR-grade water. 40 cycles of PCR were performed, followed by melting curve analysis. Concentration data were converted to mass using genomic molecular weights determined using the following approximations: molecular weight (MW) of ssDNA genome = genome length (bases) × 303.7 g/mol + 79 g/mol; MW of dsDNA genome = genome length (base pairs) × 607.4 g/mol + 157.9 g/mol.

### 16S rRNA library preparation, sequencing, and bioinformatics

16S sequencing libraries were generated by two-step PCR for each sample. In the first step, V1-V3 loops were amplified using custom adapter primers composed of universal 16S primers ‘27F’ and ‘534R’ and Illumina Nextera indexing adapter sequences. Adapter PCR was done in 25 μL reactions containing 11.5 μL of template, 0.5 μL of each primer at 10 μΜ, and 12.5 μL of KAPA HiFi HotStart ReadyMix. 25 cycles of PCR were performed under the following conditions: denaturation at 95 °C for 30 s, annealing at 55 °C for 30 s, and extension at 72 °C for 30 s. PCR products were purified with 20 μL AMPureXP beads and eluted into 50 μL of 10 mM Tris pH 8.5. In the second step, Illumina Nextera XT indices were added by PCR in 50 μL reactions containing 5 μL of product from adapter PCR, 5 μL of Index 1, 5 μL of Index 2, 25 μL of KAPA HiFi HotStart ReadyMix, and 10 μL of water. 8 cycles of PCR were conducted under the same conditions as step 1. Indexed samples were purified with 56 μL of AMPureXP beads, eluted into 25 μL of 10 mM Tris pH 8.5, quantified with a Qubit dsDNA HS kit, normalized and pooled for multiplexing. Final library QC was done using an Agilent TapeStation dsDNA 1000 bp kit. The final libraries were sequenced on an Illumina MiSeq with PE300 V3 chemistry at UCSB’s Biological Nanostructures Laboratory (BNL) sequencing core.

Paired-end reads were uploaded to the Quantitative Insights Into Microbial Ecology Amazon Web Services Amazon Machine Image (QIIME AWS AMI) (AMI ID: ami-1918ff72, “qiime-191”) [34]. Initial quality analysis was performed with FastQC. Reads were quality controlled by trimming and quality filtering with trimmomatic using default settings [35]. Read joining was performed with QIIME’s joining script (*join_paired_ends.py*), using the fastq-join algorithm with default settings. Joined reads were fed into the open operational taxonomic unit (OTU) picking pipeline (*pick_open_reference_otus.py)* using default settings. Taxonomy was assigned using the SILVA128 16S reference database clustered at the 97% identity threshold [36]. The final Biological Observation Matrix (BIOM) table (without PyNAST alignment failures) and metadata mapping files were imported into RStudio using the phyloseq package for downstream analyses [37]. Samples were sorted from controls, taxonomy was summarized by agglomerating at the family level, and absolute OTU abundance was converted to relative abundance per sample and averaged within sample type (skin vs. wound) within each study (PS1 and PS2). PS1 and PS2 phyloseq objects were converted to table format and filtered to remove any taxa with relative abundance less than 2%, and plotted with ggplot2 [38].

### VLP-enriched library preparation, sequencing, and bioinformatics

DNA from VLP-enriched samples was amplified by random hexamer-primed multiple strand displacement amplification (GenomiPhi V3, GE Healthcare), following the manufacturer’s protocol. Amplified DNA was purified with 40 μL of AMPureXP beads and eluted into 15 μL of 10 mM Tris pH 8.5. Amplified samples were normalized to 0.2 ng/uL and prepared for shotgun sequencing with the Nextera XT kit and Nextera XT indices, as described by the manufacturer. Indexed samples were quantified with a Qubit dsDNA HS kit, normalized, and pooled. Final library QC was done using Agilent TapeStation dsDNA 5000 bp and 1000 bp kits. Final libraries were sequenced on an Illumina MiSeq with PE150 (PS1) or PE300 (PS2) chemistry, at the UC Davis Genome Center.

Paired-end reads were uploaded to a custom AWS AMI (AMI ID: ami-19acbf62, “Chen Lab VMM Basic Image 1.1”) for bioinformatic processing. Initial quality analysis was performed with FastQC. Reads were quality controlled by trimming and quality filtering with trimmomatic using default settings [35]. All read mapping steps were performed with Bowtie2 using the –sensitive and –non-deterministic settings, with mapping summaries printed to file [39]. Reads were first mapped to the human genome (GRCh38.p13, NCBI accession: GCF_000001405.39). Reads that did not map to the human genome were collected and known viral lab contaminants were removed by mapping to M13 and fd genomes. Remaining reads were mapped to the Joint Genome Institute’s IMG/VR database (IMG_VR_2018-07-01_4) [30], currently the largest public database of viral metagenomes. Overall alignment rates were extracted from the mapping summaries, assembled into tables, imported to RStudio, and plotted with ggplot2 [38]. T-tests were performed with ggpubr.

## Additional file

Additional file 1: **Figure S1.** Graphical schematic of fractionation method. **Figure S2.** Additional comparisons of VLP-enriched DNA composition from clinical skin and wound swabs using kit-based extraction (PS1) and the method described here (PS2). VLP-enriched DNA was shotgun sequenced and mapped to the IMG/VR viral metagenome database (A) or the human genome (B). Means are compared by *t*-tests. Although PS2 samples contained more human DNA than PS1, the greater fraction of viral reads (Fig. 6b) also translated to a greater absolute number of reads mapping to IMG/VR. (PDF 702 kb)

## Data Availability

Data generated and analysed during the current study are available from the corresponding author upon reasonable request.

## Abbreviations

AWS AMI: Amazon Web Services Amazon Machine Image
CFU: Colony-forming unit
CTAB: Cetyltrimethylammonium bromide
gDNA: Genomic DNA
IMG/VR: Integrated Microbial Genomes viral analysis database
LB: Luria broth
MW: Molecular weight
NGS: Next-generation sequencing
OD: Optical density
OTU: Operational taxonomic unit
PS1: Pilot study 1, using a standard kit-based extraction
PS2: Pilot study 2, using the novel sample preparation
QIIME: Quantitative Insights Into Microbial Ecology software
qPCR: quantitative polymerase chain reaction
SDS: Sodium dodecyl sulfate
VLP: Virus-like particle
