# Supplementary material for: Improved single-swab sample preparation for recovering bacterial and phage DNA from human skin and wound microbiomes
Source: BMC Microbiol. 2019 Sep 5;19:214. doi: 10.1186/s12866-019-1586-4 (PMC6729076; doi:10.1186/s12866-019-1586-4)
Supplement: Supplementary file 1 — Figure S1. Graphical schematic of fractionation method. Figure S2. Additional comparisons of VLP-enriched DNA composition from clinical skin and wound swabs using kit-based extraction (PS1) and the method described here (PS2). VLP-enriched DNA was shotgun sequenced and mapped to the IMG/VR viral metagenome database (A) or the human genome (B). Means are compared by t-tests. Although PS2 samples contained more human DNA than PS1, the greater fraction of viral reads (Fig. 6b) also translated to a greater absolute number of reads mapping to IMG/VR. (PDF 702 kb) [file 12866_2019_1586_MOESM1_ESM.pdf]

Supplementary Information

S. Verbanic, C. Y. Kim, J. M. Deacon, I. A. Chen

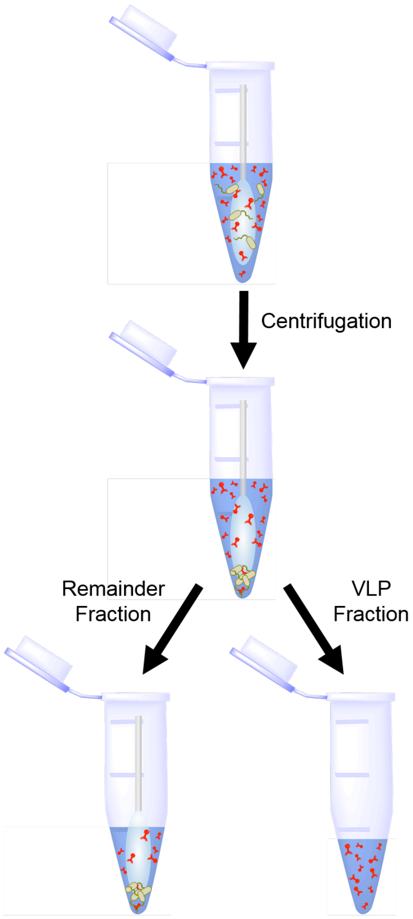

Supplemental Figure S1. Graphical schematic of fractionation method.

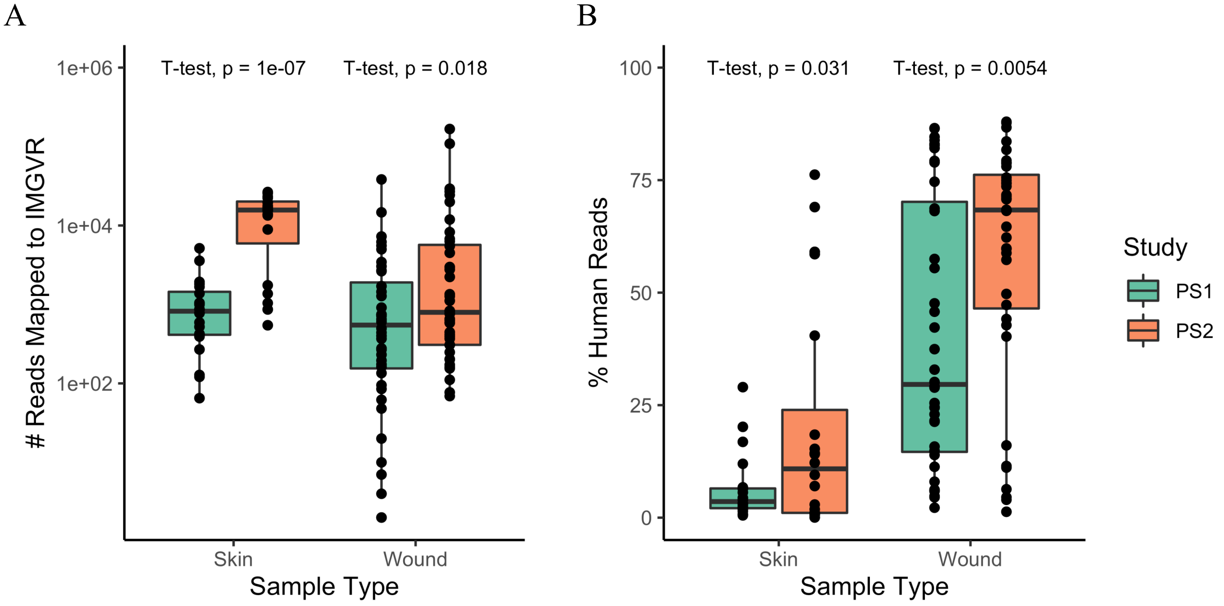

Supplemental Figure S2. Additional comparisons of VLP-enriched DNA composition from clinical skin and wound swabs using kit-based extraction (PS1) and the method described here (PS2). VLP-enriched DNA was shotgun sequenced and mapped to the IMG/VR viral metagenome database (A) or the human genome (B). Means are compared by *t*-tests. Although PS2 samples contained more human DNA than PS1, the greater fraction of viral reads (Figure 6B) also translated to a greater absolute number of reads mapping to IMG/VR.
